# Moderate weight change following diabetes diagnosis and 10 year incidence of cardiovascular disease and mortality

**DOI:** 10.1007/s00125-019-4886-1

**Published:** 2019-05-07

**Authors:** Jean Strelitz, Amy L. Ahern, Gráinne H. Long, Matthew J. L. Hare, Greg Irving, Clare E. Boothby, Nicholas J. Wareham, Simon J. Griffin

**Affiliations:** 1 MRC Epidemiology Unit, Institute of Metabolic Science, Cambridge Biomedical Campus, University of Cambridge School of Clinical Medicine, Box 285, Cambridge, CB2 0QQ UK; 2 AstraZeneca Pharmaceuticals, Cambridge, UK; 3 Departments of Endocrinology, Diabetes and Vascular Medicine, Monash Health, Melbourne, VIC Australia; 4 Primary Care Unit, Institute of Public Health, University of Cambridge School of Clinical Medicine, Cambridge, UK

**Keywords:** Cardiovascular disease, Diabetes, Epidemiology, Weight loss

## Abstract

**Aims/hypothesis:**

Adults with type 2 diabetes are at high risk of developing cardiovascular disease (CVD). Evidence of the impact of weight loss on incidence of CVD events among adults with diabetes is sparse and conflicting. We assessed weight change in the year following diabetes diagnosis and estimated associations with 10 year incidence of CVD events and all-cause mortality.

**Methods:**

In a cohort analysis among 725 adults with screen-detected diabetes enrolled in the Anglo–Danish–Dutch Study of Intensive Treatment in People with Screen-Detected Diabetes in Primary Care (ADDITION)–Cambridge trial, we estimated HRs for weight change in the year following diabetes diagnosis and 10 year incidence of CVD (*n* = 99) and all-cause mortality (*n* = 95) using Cox proportional hazards regression. We used linear regression to estimate associations between weight loss and CVD risk factors. Models were adjusted for age, sex, baseline BMI, smoking, occupational socioeconomic status, cardio-protective medication use and treatment group.

**Results:**

Loss of ≥5% body weight in the year following diabetes diagnosis was associated with improvements in HbA_1c_ and blood lipids and a lower hazard of CVD at 10 years compared with maintaining weight (HR 0.52 [95% CI 0.32, 0.86]). The associations between weight gain vs weight maintenance and CVD (HR 0.41 [95% CI 0.15, 1.11]) and mortality (HR 1.63 [95% CI 0.83, 3.19]) were less clear.

**Conclusions/interpretation:**

Among adults with screen-detected diabetes, loss of ≥5% body weight during the year after diagnosis was associated with a lower hazard of CVD events compared with maintaining weight. These results support the hypothesis that moderate weight loss may yield substantial long-term CVD reduction, and may be an achievable target outside of specialist-led behavioural treatment programmes.

**Electronic supplementary material:**

The online version of this article (10.1007/s00125-019-4886-1) contains peer-reviewed but unedited supplementary material, which is available to authorised users.

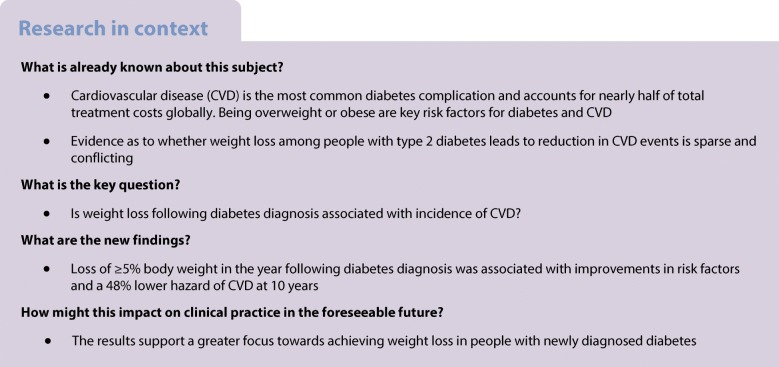



## Introduction

Adults with type 2 diabetes are at high risk of developing cardiovascular disease (CVD) [1]. CVD is the most common and most costly diabetes complication, accounting for nearly half of total treatment costs globally [2, 3]. Weight loss has been shown to improve CVD risk factors such as HbA_1c_, blood lipids and BP among people with type 2 diabetes [4–6] and may also lead to diabetes remission [7]. However, while improvements in these risk factors have been demonstrated in short-term studies [4, 8], few studies have assessed CVD events.

The existing literature on change in weight and CVD among adults with diabetes is limited, and studies have shown inconclusive and inconsistent associations of weight loss and CVD risk [9–12]. In the Action for Health in Diabetes (Look AHEAD) randomised trial among obese adults with clinically diagnosed diabetes of 7 years’ duration, the behavioural intervention assessed in the trial did not appear to reduce CVD incidence [6], although participants who lost at least 10% of their body weight in 1 year had a 21% lower 10 year risk of CVD compared with those with stable weight or weight gain [10]. However, weight loss observed among trial participants may not be representative of what would be achievable in the general population in the absence of an intensive intervention programme. Furthermore, as population-based screening for type 2 diabetes has become increasingly common, understanding the impact of weight changes early in the diabetes disease trajectory is needed to inform clinical practice at the time of diagnosis, when patients may be more receptive to care [13].

Considering the challenge of controlling CVD risk factors among individuals with established diabetes and the risks associated with intensification of therapy [14, 15], the year following diagnosis may be a critical time to establish healthy behaviours that lead to long-term comorbidity reduction. Weight loss during this period may result in a legacy effect, whereby improvements in risk factors early in the course of the disease yield long-term CVD benefits. In the UK Prospective Diabetes Study (UKPDS), intensive glucose control following diabetes diagnosis was associated with lower risk of CVD 10 years after the trial, even though differences in glucose control were not maintained after the end of the trial [16]. The Diabetes Care in General Practice trial of people with a new diagnosis of diabetes showed no association between 13 year CVD incidence and weight loss achieved through a 6 year intensive intervention [17]. However, this study had a relatively small sample size (*N* = 444) and participants had to survive until the end of the 6 year intervention period in order to be followed up for CVD, which might have resulted in a healthier study population relative to the target population [18]. The Retrospective Study of Cardiovascular Events Related to the Use of Glucose-Lowering Drug Treatment in Primary Care (ROSE) cohort study of individuals in Swedish primary care (*N* = 8486) showed that weight loss in the 18 months following diabetes diagnosis had no protective association with CVD events during a median follow-up time of 4.6 years; however, weight gain was associated with 63% higher hazard of CVD [12]. Given the mixed results of studies of weight loss and CVD and the limited generalisability of results from intensive weight loss trials, additional population-based research is needed to characterise the impacts of weight loss after diabetes diagnosis on long-term CVD incidence.

Using data from a population-based study of screening for type 2 diabetes, the Anglo–Danish–Dutch Study of Intensive Treatment in People with Screen-Detected Diabetes in Primary Care (ADDITION)–Cambridge [19], we assessed the association of weight change in the year following diabetes diagnosis and 10 year CVD and mortality incidence, and the impact of weight change on CVD risk factors (BP, blood lipid and HbA_1c_ levels) at 1 year and 5 years following diagnosis.

## Methods

ADDITION–Cambridge (ISRCTN86769081) is a pragmatic cluster-randomised trial comparing multifactorial intervention vs routine care among people with screen-detected diabetes from 49 general practices (GPs) in eastern England [19]. The present study is an observational analysis of the trial cohort. A validated risk score was used to identify eligible individuals aged 40–69 years who were at high risk of diabetes, using the electronic records of participating GPs [20]. From 2002 to 2006, 33,539 high-risk individuals were invited to attend a stepwise screening programme; 24,654 (74%) took part [21]. During screening, 867 participants were diagnosed with type 2 diabetes using the 1999 WHO criteria [22] and all consented to enrol in the study. The GPs were cluster-randomised to intensive treatment (*n* = 26) or routine care (*n* = 23). In the routine care group, GPs were advised to follow current UK guidelines for diabetes management [23–25]. Intensive treatment included more frequent consultations, provision of educational materials and GP-based academic-detailing sessions encouraging earlier use of medication to improve control of risk factors. The intervention did not include behavioural treatment. Written informed consent was obtained from all participants and ethics approval was obtained from the research ethics committees in Cambridge (ref. 01/063), Huntingdonshire (ref. 00/609), Peterborough and Fenland (ref. P01/95), West Essex (ref. 1511–0103), North and Mid Essex (ref. MH395 MREC02/5/54), West Suffolk (ref. 03/002), and Hertfordshire and Bedfordshire (ref. EC03623) Local Research Ethics Committees and the Eastern Multi-Centre Research Ethics Committee (ref. 02/5/54).

### Measurements

Anthropometric, biochemical, clinical and questionnaire-based measures were taken at the time of diabetes diagnosis (baseline) and after 1 and 5 years [19]. Sociodemographic information (age, sex, occupation and ethnicity), smoking status and prescribed pharmacological treatment were self-reported via standardised questionnaires. Information on pharmacological treatment at 5 years was supplemented using GP electronic records. Socioeconomic status (SES) was defined according to the Registrar General’s occupation-based classification: ‘professional, managerial and technical’, ‘skilled-manual and non-manual’ and ‘partly skilled or unskilled’ [26]. Clinical and anthropometric measures were performed by trained staff, according to standard procedures [19].

### CVD and mortality outcomes

The outcomes of interest were CVD events and all-cause mortality. The composite CVD outcome included cardiovascular mortality, non-fatal myocardial infarction, non-fatal stroke, non-traumatic amputation, and revascularisation (both invasive cardiovascular and peripheral vascular procedures). Incidence of CVD events and mortality was ascertained from the date of diabetes diagnosis until 31 December 2014. Participants were flagged using National Health Service (NHS) patient numbers for mortality surveillance by the Office for National Statistics. Possible CVD outcomes were identified via searches of GP notes, hospital discharge summaries, hospital notes, electrocardiograms, laboratory results, death certificates, autopsies and the Myocardial Ischaemia National Audit Project (MINAP) [27]. All events were independently adjudicated using standardised case report forms.

### Statistical analysis

Of the 867 adults enrolled in the ADDITION–Cambridge study, we were unable to include 137 for whom we could not determine weight change, as they were missing data on weight measurements at baseline or 1 year. We also excluded individuals who had a CVD event in the first year in the study (*n* = 5), as this was when weight change was measured. Therefore, this study included 725 participants with 99 CVD events. Mortality analyses also excluded deaths occurring within 1 year after weight change was assessed (*n* = 2), as weight loss might have been due to underlying disease; this left a total of 95 all-cause mortalities during the study period.

We assessed predictors of missing weight information by comparing distributions of factors measured at baseline between individuals who were and who were not missing weight measurements.

The proportion of weight change was determined by subtracting weight at baseline from weight at 1 year, then dividing by weight at baseline. We defined weight change categorically as >2% gain, maintained weight (≤2% gain or <2% loss), ≥2% to <5% loss, ≥5% to <10% loss, and ≥10% loss. These categories were chosen to examine the recommended weight loss target of 5–10% [28, 29] and compare it against greater or lesser weight loss, while separating those who gained weight from those who maintained their baseline weight.

#### Weight change, CVD events and all-cause mortality

We used Cox proportional hazards regression to estimate HRs for category of weight change, 10 year incidence of CVD events and 10 year all-cause mortality. Due to the small numbers of CVD events among participants who lost the most weight, we combined the ≥5% to <10% loss and ≥10% loss categories.

The timescale was the number of days since diabetes diagnosis. Person-time at risk began 1 year after diabetes diagnosis and ended at the time of the event, death or 31 December 2014. We assessed the proportional hazards assumption by modelling an interaction term between the natural log of time and each covariate, which indicated no departures from proportional hazards. All models adjusted for confounders identified using a directed acyclic graph [30]: age at baseline (continuous), sex (female, male), trial group (intensive treatment, routine care), baseline occupational SES (‘professional, managerial and technical’, ‘skilled-manual and non-manual’ and ‘partly skilled or unskilled’), BMI at baseline (continuous, coded as a quadratic term), cigarette-smoking status at 1 year (current, former, never), and use of antihypertensive (yes, no), glucose-lowering (yes, no) and lipid-lowering (yes, no) medication at 1 year. Clustering of individuals within GPs was accounted for using a robust cluster variance estimator.

To assess heterogeneity of associations by age, we modelled an interaction term between weight change and age at diagnosis. As past research has shown negative impacts of weight loss on mortality among older adults [31–33], we performed separate analyses to estimate the associations of interest among those aged ≥65 years at the time of diabetes diagnosis. We were unable to stratify by age at diagnosis <65 years owing to the small number of cases in this age group.

In a sensitivity analysis, we used multiple imputation by chained equations [34] to estimate associations among the full cohort including the 137 individuals with missing weight information. The imputation models included covariates for weight change category; sex; occupational SES; baseline BMI; smoking; treatment group; antihypertensive, glucose-lowering and lipid-lowering medication use at 1 year; outcome status; and the Nelson–Aalen estimate of cumulative hazard. We generated 20 imputed datasets and used these to estimate HRs in the full cohort. Separate imputation models were fit where the outcome was all-cause mortality. We also assessed the robustness of our results to changes in weight after the first year in the study by adjusting for weight change from year 1 to year 5. Additional sensitivity analyses excluded individuals with a history of myocardial infarction or stroke prior to diagnosis of diabetes (*n* = 77); separate analyses excluded revascularisation and amputations from the composite CVD outcome (*n* = 37) in order to estimate HRs for a composite of stroke, myocardial infarction or cardiovascular death. To evaluate the association of weight change and CVD occurring in the presence of the competing risk of non-CVD mortality, we estimated subdistribution HRs [35].

#### Weight loss and CVD risk factors

We used linear regression models to estimate the associations between percentage weight loss in the year following diabetes diagnosis and CVD risk factors (HbA_1c_, systolic and diastolic BP, triacylglycerols, total cholesterol and LDL-cholesterol) at 1 year and 5 years. Percentage weight change categories were defined as above.

The adjustment set was the same as in the proportional hazards models, except that each model was adjusted for relevant medication use in the year the outcome was measured (e.g. glucose-lowering agents for HbA_1c_, antihypertensive medication for BP, and lipid-lowering medication for lipid levels), rather than adjusting for each type of medication use. Separate analyses were stratified by medication use at 1 year and 5 years to assess possible heterogeneity of the associations. Sensitivity analyses were conducted: (1) using multiple imputation by chained equations [34] to estimate the associations of percentage weight change and the CVD risk factors among the full cohort; [2] by adjusting for baseline values of the risk factors.

## Results

Of the 99 first incident CVD events, 36 were revascularisations, 24 were strokes, 21 were CVD deaths, 17 were myocardial infarctions and one was an amputation. Of the 95 incident deaths during the study period, 31 were related to CVD, 45 to cancer and 19 to another cause. Mean follow-up time was 9.8 years.

Of the 725 study participants with non-missing weight information and no CVD event during the first year in the study, 62% were male, 97% were white and the mean age at diagnosis of diabetes was 61 years. At baseline 34.8% had a professional occupation, 23.0% had a skilled occupation and 42.3% had an unskilled occupation. Mean weight change (SD) during the study period was −3.6 kg (5.5 kg) at 1 year (Table 1) and −4.1 kg (7.1 kg) at 5 years (not shown). Use of lipid-lowering and glucose-lowering medication increased throughout the study period (Table 1). As distributions of weight changes and CVD risk factors were similar between men and women (electronic supplementary material [ESM] Table 1), all participants were analysed together. CVD risk factors largely improved across the study period. Those who lost ≥5% body weight had somewhat larger decreases in systolic BP at 1 year and 5 years compared with the other groups (Table 2). After stratifying by category of weight change in the first year in the study, the mean weight at 5 years in the study was similar to the mean weight at 1 year in the study (ESM Table 2).

**Table 1:** Characteristics of individuals with type 2 diabetes at the time of diagnosis (baseline) and 1 year later by weight change category in the year following diabetes diagnosis

Characteristic	Full cohort (*N* = 725)		Gained >2% weight (*n* = 79)		Maintained weight^a^ (*n* = 222)		Lost ≥2% to <5% weight (*n* = 183)		Lost ≥5% weight (*n* = 241)	
	Baseline	1 year	Baseline	1 year	Baseline	1 year	Baseline	1 year	Baseline	1 year
Sex, *n* (%)										
Female	279 (38.5)	–	30 (38.0)	–	71 (32.0)	–	64 (35.0)	–	114 (47.3)	–
Male	446 (61.5)	–	49 (62.0)	–	151 (68.0)	–	119 (65.0)	–	127 (52.7)	–
Missing	0 (0.0)	–	0 (0.0)	–	0 (0.0)	–	0 (0.0)	–	0 (0.0)	–
Age, years	61.1 (7.1)	–	60.8 (7.1)	–	60.4 (7.5)	–	61.0 (7.3)	–	61.8 (6.4)	–
Missing, *n* (%)	0 (0.0)	–	0 (0.0)	–	0 (0.0)	–	0 (0.0)	–	0 (0.0)	–
BMI, kg/m^2^	33.4 (5.6)	32.2 (5.5)	32.3 (6.3)	33.8 (6.6)	32.8 (5.4)	32.8 (5.4)	33.4 (5.4)	32.4 (5.2)	34.2 (5.6)	30.9 (5.1)
Missing, *n* (%)	1 (0.1)	0 (0.0)	0 (0.0)	0 (0.0)	0 (0.0)	1 (0.5)	1 (0.5)	0 (0.0)	0 (0.0)	0 (0.0)
Weight, kg	94.6 (17.6)	91.0 (17.4)	91.5 (18.6)	95.8 (18.9)	94.3 (17.5)	94.0 (17.5)	95.0 (16.1)	91.9 (15.6)	95.7 (18.3)	86.1 (16.8)
Missing, *n* (%)	0 (0.0)	0 (0.0)	0 (0.0)	0 (0.0)	0 (0.0)	0 (0.0)	0 (0.0)	0 (0.0)	0 (0.0)	0 (0.0)
Weight change from baseline, kg	–	−3.6 (5.5)	–	4.3 (2.0)	–	−0.27 (1.0)	–	−3.1 (0.9)	–	−9.6 (4.7)
Smoking, *n* (%)										
Current	120 (16.6)	106 (14.8)	12 (15.2)	9 (11.5)	34 (15.3)	27 (12.4)	31 (16.9)	32 (17.6)	43 (17.8)	38 (16.0)
Former	337 (46.5)	343 (48.0)	27 (34.2)	29 (37.2)	124 (55.9)	127 (58.3)	86 (47.0)	85 (46.7)	100 (41.5)	102 (43.0)
Never	268 (37.0)	266 (37.2)	40 (50.6)	40 (51.3)	64 (28.8)	64 (29.4)	66 (36.1)	65 (35.7)	98 (40.7)	97 (40.9)
Missing	0 (0.0)	10 (1.4)	0 (0.0)	1 (1.3)	0 (0.0)	4 (1.8)	0 (0.0)	1 (0.5)	0 (0.0)	4 (1.7)
Alcohol										
Units/week	7.5 (10.8)	6.6 (9.8)	10.0 (13.6)	9.6 (13.5)	9.0 (11.8)	8.4 (11.1)	7.1 (10.6)	5.9 (8.6)	5.6 (8.3)	4.6 (7.3)
Missing, *n* (%)	11 (1.5)	17 (2.3)	1 (1.3)	2 (2.5)	4 (1.8)	5 (2.3)	3 (1.6)	5 (2.7)	3 (1.2)	5 (2.1)
Prescribed medication use, *n* (%)										
Glucose-lowering										
Yes	2 (0.3)	216 (30.6)	1 (1.3)	31 (40.8)	0 (0.0)	63 (29.4)	1 (0.5)	60 (33.7)	0 (0.0)	62 (26.2)
No	723 (99.7)	489 (69.4)	78 (98.7)	45 (59.2)	222 (100.0)	151 (70.6)	182 (99.5)	118 (66.3)	241 (100.0)	175 (73.8)
Missing	0 (0.0)	20 (2.8)	0 (0.0)	3 (3.8)	0 (0.0)	8 (3.6)	0 (0.0)	5 (2.7)	0 (0.0)	4 (1.7)
Antihypertensive										
Yes	414 (57.1)	485 (68.8)	51 (64.6)	56 (73.7)	121 (54.5)	136 (63.6)	111 (60.7)	130 (73.0)	131 (54.4)	163 (68.8)
No	311 (42.9)	220 (31.2)	28 (35.4)	20 (26.3)	101 (45.5)	78 (36.4)	72 (39.3)	48 (27.0)	110 (45.6)	74 (31.2)
Missing	0 (0.0)	20 (2.8)	0 (0.0)	3 (3.8)	0 (0.0)	8 (3.6)	0 (0.0)	5 (2.7)	0 (0.0)	4 (1.7)
Lipid-lowering										
Yes	173 (23.9)	462 (65.5)	18 (22.8)	51 (67.1)	58 (26.1)	123 (57.5)	48 (26.2)	128 (71.9)	49 (20.3)	160 (67.5)
No	552 (76.1)	243 (34.5)	61 (77.2)	25 (32.9)	164 (73.9)	91 (42.5)	135 (73.8)	50 (28.1)	192 (79.7)	77 (32.5)
Missing	0 (0.0)	20 (2.8)	0 (0.0)	3 (3.8)	0 (0.0)	8 (3.6)	0 (0.0)	5 (2.7)	0 (0.0)	4 (1.7)

Data are from a prospective cohort analysis of participants recruited in ADDITION–Cambridge between 2002 and 2014

Data are presented as mean (SD) except where otherwise stated

^a^Defined as ≤2% gain or <2% loss

**Table 2:** Distribution of CVD risk factors and changes in CVD risk factors between baseline and 1 year and 5 years post diagnosis, by category of change in weight in the year following diabetes diagnosis

CVD risk factor	Full cohort (*N* = 725)			Gained >2% weight (*n* = 79)			Maintained^a^ (*n* = 222)			Lost ≥2% to <5% weight (*n* = 183)			Lost ≥5% weight (*n* = 241)		
	Baseline	1 year	5 years	Baseline	1 year	5 years	Baseline	1 year	5 years	Baseline	1 year	5 years	Baseline	1 year	5 years
HbA_1c_, median (Q1, Q3)															
mmol/mol	50.8 (45.4, 60.7)	46.4 (42.1, 50.8)	51.9 (46.4, 57.4)	50.8 (44.3, 86.9)	48.6 (44.3, 57.4)	54.1 (46.4, 59.6)	49.7 (44.3, 57.4)	48.6 (43.2, 54.1)	51.9 (47.5, 58.5)	50.8 (45.4, 60.7)	46.4 (42.1, 50.8)	51.9 (47.5, 57.4)	50.8 (45.4, 61.7)	43.2 (39.9, 46.4)	48.6 (45.4, 56.3)
%	6.8 (6.3, 7.7)	6.4 (6.0, 6.8)	6.9 (6.4, 7.4)	6.8 (6.2, 10.1)	6.6 (6.2, 7.4)	7.1 (6.4, 7.6)	6.7 (6.2, 7.4)	6.6 (6.1, 7.1)	6.9 (6.5, 7.5)	6.8 (6.3, 7.7)	6.4 (6.0, 6.8)	6.9 (6.5, 7.4)	6.8 (6.3, 7.8)	6.1 (5.8, 6.4)	6.6 (6.3, 7.3)
Missing, *n*	18	10	61	0	1	7	11	4	24	6	1	11	1	4	19
BP, mmHg															
Systolic	141.6 (19.7)	136.1 (18.4)	135.0 (15.8)	142.1 (21.4)	139.9 (18.9)	135.0 (16.1)	141.9 (18.8)	138.1 (18.4)	135.9 (14.4)	137.4 (17.2)	134.6 (18.4)	133.7 (16.5)	144.3 (21.3)	134.3 (17.8)	135.2 (16.6)
Diastolic	81.7 (10.3)	78.7 (9.6)	75.3 (9.7)	82.4 (11.7)	80.5 (9.4)	75.4 (9.9)	82.2 (10.2)	80.2 (10.1)	76.0 (9.4)	80.9 (10.1)	78.9 (9.9)	75.5 (10.8)	81.7 (10.1)	76.6 (8.5)	74.4 (8.9)
Missing, *n*	2	2	51	1	1	7	0	0	19	1	1	10	0	0	15
Lipids, mmol/l															
Total cholesterol	5.4 (1.1)	4.5 (1.0)	4.2 (0.9)	5.3 (1.2)	4.4 (1.0)	4.1 (0.9)	5.3 (1.1)	4.7 (1.0)	4.3 (0.9)	5.4 (1.2)	4.5 (1.0)	4.1 (0.9)	5.5 (1.1)	4.4 (0.9)	4.1 (0.8)
Missing, *n*	15	3	59	0	0	7	6	1	22	7	0	12	2	2	18
LDL-cholesterol	3.3 (1.0)	2.5 (0.8)	2.1 (0.7)	3.2 (1.0)	2.4 (0.8)	2.1 (0.8)	3.2 (0.9)	2.7 (0.9)	2.2 (0.8)	3.3 (1.1)	2.5 (0.8)	2.0 (0.7)	3.4 (0.9)	2.5 (0.8)	2.0 (0.7)
Missing, *n*	43	27	96	4	0	10	18	16	36	12	6	23	9	5	27
HDL-cholesterol	1.2 (0.3)	1.2 (0.3)	1.3 (0.3)	1.2 (0.4)	1.2 (0.4)	1.3 (0.3)	1.2 (0.3)	1.2 (0.3)	1.3 (0.3)	1.2 (0.4)	1.2 (0.4)	1.3 (0.3)	1.2 (0.3)	1.3 (0.3)	1.4 (0.4)
Missing, *n*	15	3	74	0	0	10	6	1	28	7	0	14	2	2	22
Triacylglycerols, median (Q1, Q3)	1.8 (1.2, 2.5)	1.6 (1.2, 2.3)	1.7 (1.2, 2.3)	1.6 (1.0, 2.7)	1.8 (1.2, 2.4)	1.5 (1.0, 2.2)	1.8 (1.2, 2.4)	1.9 (1.3, 2.6)	1.8 (1.4, 2.5)	1.9 (1.2, 2.6)	1.7 (1.2, 2.3)	1.7 (1.2, 2.4)	1.8 (1.3, 2.4)	1.4 (1.0, 2.0)	1.5 (1.0, 2.3)
Missing, *n*	16	3	69	0	0	8	7	1	25	7	0	14	2	2	22
Changes from baseline															
HbA_1c_															
mmol/mol	–	−9.1 (16.8)	−3.0 (19.0)	–	−12.8 (24.8)	−10.0 (26.9)	–	−6.7 (18.8)	−1.2 (19.0)	–	−7.6 (13.5)	−2.2 (17.1)	–	−11.0 (13.2)	−2.8 (16.8)
%	–	−0.8 (1.5)	−0.3 (1.7)	–	−1.1 (2.3)	−0.9 (2.5)	–	−0.6 (1.7)	−0.1 (1.7)	–	−0.7 (1.2)	−0.2 (1.6)	–	−1.0 (1.2)	−0.3 (1.5)
BP, mmHg															
Systolic	–	−5.5 (18.7)	−6.3 (20.1)	–	−2.2 (21.4)	−6.1 (21.7)	–	−4.0 (17.9)	−5.9 (20.1)	–	−2.8 (17.3)	−3.2 (18.9)	–	−10.1 (18.7)	−9.4 (20.5)
Diastolic	–	−3.0 (9.8)	−6.4 (11.2)	–	−2.0 (11.7)	−6.7 (12.6)	–	−2.0 (10.0)	−6.1 (11.0)	–	−2.0 (9.7)	−5.5 (11.1)	–	−5.1 (9.0)	−7.5 (11.1)
Lipids, mmol/l															
Total cholesterol	–	−0.8 (1.1)	−1.2 (1.1)	–	−0.9 (1.2)	−1.2 (1.2)	–	−0.6 (1.0)	−1.1 (1.1)	–	−0.8 (1.1)	−1.3 (1.2)	–	−1.1 (1.1)	−1.4 (1.1)
LDL-cholesterol	–	−0.8 (1.0)	−1.2 (1.0)	–	−0.8 (1.0)	−1.1 (1.1)	–	−0.6 (0.9)	−1.1 (0.9)	–	−0.8 (1.0)	−1.4 (1.1)	–	−1.0 (1.0)	−1.4 (0.9)
HDL-cholesterol	–	0.0 (0.2)	0.1 (0.3)	–	−0.0 (0.2)	0.1 (0.3)	–	0.0 (0.2)	0.1 (0.2)	–	0.0 (0.2)	0.1 (0.2)	–	0.1 (0.2)	0.2 (0.3)
Triacylglycerols	–	−0.2 (1.2)	−0.2 (1.2)	–	−0.1 (1.4)	−0.3 (1.3)	–	0.0 (1.1)	−0.1 (1.2)	–	−0.1 (1.0)	−0.2 (1.1)	–	−0.5 (1.1)	−0.4 (1.3)

Data are from a prospective cohort analysis of participants recruited in ADDITION–Cambridge between 2002 and 2014

Data are presented as mean (SD) except where otherwise stated

^a^Defined as ≤2% gain or <2% loss

Q1, quartile; Q3, quartile 3

Missing weight information at baseline or 1 year was associated with SES and smoking: unskilled workers were more likely to have missing weight compared with professional workers, and non-smokers were less likely to have missing weight compared with current smokers (ESM Table 3).

### Weight change, 10 year CVD events and all-cause mortality

Losing ≥5% of body weight was associated with a lower hazard of 10 year CVD events compared with maintaining weight (HR 0.52 [95% CI 0.32, 0.86]). Associations between weight gain and 10 year CVD incidence (HR 0.41 [95% CI 0.15, 1.11]) and all-cause mortality (HR 1.63 [95% CI 0.83, 3.19]) were less apparent. There were no associations between weight loss and all-cause mortality (Table 3). The associations were similar after adjusting for changes in weight between 1 and 5 years in the study (ESM Table 4). Results from analyses with multiple imputation of missing information also showed similar associations (ESM Table 5). Analyses excluding participants with self-reported history of CVD were also similar (ESM Table 6), as were analyses excluding revascularisations and amputations (ESM Table 7). In the subdistribution hazards analysis, the associations with CVD were unchanged (ESM Table 8).

**Table 3:** HRs for the associations of change in weight in the year following diabetes diagnosis and 10 year incidence of CVD and mortality

Sample	10 year CVD incidence		10 year all-cause mortality	
	Cases/total, *n*	HR (95% CI)^a^	Cases/total, *n*	HR (95% CI)^a^
Full cohort (*N* = 725)				
Weight change				
Gained >2%	6/75	0.41 (0.15, 1.11)	13/75	1.63 (0.83, 3.19)
Maintained^b^	40/210	1.00	23/210	1.00
Lost ≥2% to <5%	26/173	0.79 (0.43, 1.46)	22/172	1.08 (0.60, 1.93)
Lost ≥5%	22/229	0.52 (0.32, 0.86)	30/228	1.12 (0.52, 2.37)
Age ≥65 years (*n* = 278)				
Weight change				
Gained >2%	4/26	0.66 (0.20, 2.15)	9/26	2.07 (0.81, 5.30)
Maintained^b^	20/78	1.00	14/78	1.00
Lost ≥2% to <5%	16/72	0.87 (0.41, 1.87)	18/72	1.32 (0.63, 2.78)
Lost ≥5%	8/84	0.39 (0.16, 0.94)	15/84	1.03 (0.37, 2.81)

Data are from a prospective cohort analysis of participants recruited in ADDITION–Cambridge between 2002 and 2014

Sample sizes comprise only cases where data are available for weight change, age, sex, baseline SES, baseline BMI, smoking at 1 year and use of antihypertensive, lipid- or glucose-lowering medication at 1 year

^a^HRs are adjusted for age, sex, baseline SES, baseline BMI, smoking at 1 year, use of antihypertensive, lipid- or glucose-lowering medication at 1 year, and trial arm

^b^Defined as ≤2% gain or <2% loss at 1 year

There was no evidence of an interaction between age at diabetes diagnosis and weight change (*p* > 0.05). Among those aged ≥65 years at the time of diabetes diagnosis, losing ≥5% body weight was associated with a lower hazard of CVD compared with maintaining weight (HR 0.39 [95% CI 0.16, 0.94]) but was not associated with all-cause mortality. Weight gain was suggestively associated with a lower hazard of CVD (HR 0.66 [95% CI 0.20, 2.15]) and a higher hazard of mortality (HR 2.07 [95% CI 0.81, 5.30]) among those aged ≥65 years, albeit the results were not statistically significant (Table 3).

### Weight change and CVD risk factors

Compared with participants who maintained weight, those who lost ≥5% body weight had lower HbA_1c_, diastolic BP and triacylglycerols at 1 year. At 5 years, these improvements were only apparent among those who had lost ≥10% weight. Total cholesterol was improved at 1 year and 5 years among those who lost ≥10% weight. Those who gained weight had suggestive improvements in total and LDL-cholesterol at 1 year but not at 5 years (Table 4). After multiple imputation of missing weight information, the observed associations were slightly stronger (ESM Table 9). After adjusting for baseline risk factor values, the results were similar (ESM Table 10).

**Table 4:** β coefficients and 95% CIs derived from multivariable linear regression models of the associations of weight change in the year following diabetes diagnosis and cardiovascular risk factors measured at 1 and 5 years after diagnosis

Risk factor	Outcomes measured at 1 year		Outcomes measured at 5 years	
	*n*	β (95% CI)^a^	*n*	β (95% CI)^a^
HbA_1c_ (mmol/mol)				
Gained >2%	74	2.33 (−0.26, 4.92)	71	−0.36 (−3.45, 2.73)
Maintained^b^	206	0	192	0
Lost ≥2% to <5%	172	−1.95 (−4.00, 0.11)	166	−1.25 (−3.67, 1.16)
Lost ≥5% to <10%	129	−4.91 (−6.54, −3.27)	124	−1.10 (−3.95, 1.74)
Lost ≥10%	96	−6.92 (−9.14, −4.71)	90	−3.72 (−6.28, −1.16)
Systolic BP, mmHg				
Gained >2%	75	0.93 (−5.22, 7.09)	71	−0.96 (−5.39, 3.47)
Maintained^b^	210	0	196	0
Lost ≥2% to <5%	172	−3.52 (−6.59, −0.45)	168	−1.47 (−4.79, 1.85)
Lost ≥5% to <10%	131	−2.65 (−7.04, 1.74)	125	1.67 (−2.80, 6.14)
Lost ≥10%	98	−4.73 (−9.17, −0.29)	92	−2.30 (−6.44, 1.83)
Diastolic BP, mmHg				
Gained >2%	75	0.11 (−2.25, 2.47)	71	−0.45 (−3.77, 2.87)
Maintained^b^	210	0	196	0
Lost ≥2% to <5%	172	−1.27 (−2.90, 0.36)	168	−0.56 (−2.36, 1.24)
Lost ≥5% to <10%	131	−2.37 (−4.03, −0.71)	125	−0.44 (−2.32, 1.44)
Lost ≥10%	98	−3.54 (−5.86, −1.23)	92	−2.41 (−4.76, −0.07)
Total cholesterol, mmol/l				
Gained >2%	75	−0.26 (−0.51, −0.01)	71	−0.11 (−0.29, 0.06)
Maintained^b^	209	0	194	0
Lost ≥2% to <5%	173	−0.09 (−0.26, 0.09)	165	−0.17 (−0.32, −0.02)
Lost ≥5% to <10%	130	−0.24 (−0.46, −0.02)	123	−0.15 (−0.32, 0.02)
Lost ≥10%	97	−0.32 (−0.49, −0.15)	92	−0.21 (−0.38, −0.04)
LDL-cholesterol, mmol/l				
Gained >2%	75	−0.25 (−0.46, −0.04)	68	−0.04 (−0.23, 0.15)
Maintained^b^	195	0	182	0
Lost ≥2% to <5%	168	−0.06 (−0.20, 0.08)	155	−0.19 (−0.33, −0.04)
Lost ≥5% to <10%	128	−0.12 (−0.25, 0.00)	117	−0.12 (−0.26, 0.02)
Lost ≥10%	96	−0.12 (−0.25, 0.00)	89	−0.15 (−0.30, 0.01)
Triacylglycerols^c^, mmol/l				
Gained >2%	75	−0.11 (−0.25, 0.04)	70	−0.17 (−0.31, −0.03)
Maintained^b^	209	0	192	0
Lost ≥2% to <5%	173	−0.10 (−0.21, 0.00)	164	−0.07 (−0.19, 0.04)
Lost ≥5% to <10%	130	−0.21 (−0.33, −0.10)	121	−0.09 (−0.23, 0.05)
Lost ≥10%	97	−0.46 (−0.59, −0.32)	90	−0.30 (−0.42, −0.17)

Data are from a prospective cohort analysis of *N* = 725 participants recruited in ADDITION–Cambridge between 2002 and 2014

^a^β coefficient values are from linear regression models adjusted for age, sex, SES, baseline BMI, smoking at 1 year, relevant medication use at 1 or 5 years, and trial arm. Coefficients represent the change in the specified outcome of interest by category of weight change, with all other variables in the model held constant

^b^Defined as ≤2% gain or <2% loss at 1 year

^c^Calculated from log_*e*_ of triacylglycerols in mmol/l

After stratifying by relevant medication use at 1 year, participants who lost ≥5% body weight had lower triacylglycerols, irrespective of lipid-lowering medication use (ESM Table 11).

## Discussion

In this study of 725 individuals with screen-detected type 2 diabetes, loss of ≥5% body weight was associated with a 48% lower 10 year hazard of CVD incidence vs maintaining weight, after adjustment for age, sex, baseline BMI, SES, smoking, medication use and trial arm. Associations were independent of changes in weight between 1 and 5 years in the study and were consistent among adults aged ≥65 years at the time of diabetes diagnosis. Loss of ≥10% body weight was associated with improvements in HbA_1c_, total cholesterol and triacylglycerols at 5 years following diagnosis, which might have contributed to the observed association of weight loss and CVD events. There was no apparent association between weight loss and all-cause mortality. Weight gain was also non-statistically significantly associated with lower hazard of CVD, but possibly higher hazard of all-cause mortality.

While extreme weight loss among people with type 2 diabetes achieved through bariatric surgery has been shown to reduce the risk of CVD [36], studies of weight loss in non-surgery populations have shown inconsistent associations with mortality and incidence of CVD [9, 37]. A Scottish observational study of weight change within 2 years of diabetes diagnosis and 5 year incidence of CVD showed no benefits of weight loss [11]. However, the magnitude of weight lost in this cohort was relatively low (mean weight change −0.7% ± 6.7%) compared with the −3.7% ± 5.7% mean weight change in the current study. The ROSE study also showed no association between reductions in BMI in the 18 months following diabetes diagnosis and 5 year CVD risk [12]. While the magnitude of weight loss in the ROSE study was comparable to that in ADDITION–Cambridge (mean BMI decrease among those who lost weight was 2.5 kg/m^2^, or 7.6% of baseline BMI), those who had lost weight had a higher BMI across the study period compared with those with unchanged weight. This was not the case in our study, where those who lost the most weight had a lower BMI across the study period compared with those who had maintained or gained weight (ESM Table 2). Additionally, the ROSE study had a relatively short follow-up (median 5 years) and did not control for antihypertensive medication use or smoking [38]. Furthermore, unintentional weight loss due to underlying disease might have masked any protective impact of weight loss on CVD.

One year weight loss of ≥10% body weight was associated with a 21% lower 10 year hazard of CVD in the Look AHEAD trial. We observed similarly strong, protective associations with ≥5% weight loss. This may be due to the fact that we targeted weight loss following diagnosis of diabetes via screening, whereas in Look AHEAD weight loss occurred, on average, 7 years after clinical diabetes diagnosis. Weight loss in the early stages of diabetes may result in a legacy effect to reduce long-term incidence of CVD, as was observed with short-term intensive glucose control in the UKPDS [16]. Weight loss was associated with improvements in HbA_1c_, lipid levels and BP at 1 year in the study. Few of these associations persisted at 5 years; however, it is possible that improvement in risk factors early on contributed to the observed lower incidence of CVD among those who lost the most weight. Furthermore, changes in BP, blood lipids and blood glucose only partially explain the mechanisms by which healthy behaviours lower CVD risk [39]. Increases in physical activity among those who lost weight might have reduced the risk of CVD through inflammatory mechanisms independently of the risk factors measured in our study.

Unintentional weight loss due to sarcopenia or cancer increases the short-term risk of mortality [40] and may be a source of unmeasured confounding in our study. To address potential confounding, we excluded deaths occurring in the year following the assessment of weight change, to reduce the likelihood that weight loss in our study was caused by disease. The protective association between weight loss and CVD risk observed in this study remained evident after restriction to participants aged ≥65 years at the time of diagnosis. We cannot definitively determine that weight loss in this study was intentional and because of healthy lifestyle changes rather than age-related or cancer-related wasting; however, the fact that we excluded deaths occurring within 1 year following the initial weight loss, and that our results were robust among older participants, supports our interpretation that weight loss was unlikely to be the result of underlying disease. Unmeasured socioeconomic or baseline health differences may also modify associations with weight loss; it is possible that weight loss may be achievable and helpful for some people but unachievable or unhealthy for others. We cannot rule out the possibility that those who lost weight might have been more willing and able to healthily lose weight compared with those who did not lose weight, or the potential for other unmeasured confounding. Therefore, our results may only be applicable to people who are willing and able to lose weight.

Compared with maintaining weight, weight gain >2% had a suggestive protective association with CVD and a suggestive adverse association with mortality. These results were not statistically significant and should be interpreted with caution, as only 75 study participants gained weight. Considering the existing literature, there is no substantiating evidence that weight gain is protective against CVD among people with type 2 diabetes. In the ROSE study, weight gain was associated with a higher hazard of 5 year all-cause mortality compared with maintaining weight [12]. Smoking cessation is associated with reduced CVD risk and weight gain; however, it is unlikely that it would have introduced confounding, as we controlled for cigarette smoking and only 17 study participants had given up smoking during the 1 year period when weight was measured. There may be heterogeneity in the associations of weight gain and CVD by baseline BMI; we adjusted for baseline BMI but we did not have a sufficient sample size to perform stratified analyses. Among those who gained weight, we might have under-ascertained CVD incidence because of the competing risk of mortality; however, as we censored participants at the time of death, person-time following a competing event was not included in the risk set. In a sensitivity analysis we estimated subdistribution HRs [35] to incorporate person-time of individuals who experienced a competing event, and the results were unchanged. Regardless, the observed associations of weight gain and CVD had poor precision and may be due to chance.

The ADDITION–Cambridge cohort was predominantly white, which limits generalisability to other populations. The majority of participants were overweight or obese at diagnosis, and although we adjusted for baseline BMI, the results would not necessarily apply to people with a normal weight or those who are underweight. We had approximately 15% missing information on weight at baseline or 1 year follow-up. Multiple imputation of missing information on weight showed generally similar associations, indicating results were robust to missing data under the assumption that missingness occurred at random, conditional on the covariates included in the imputation model. The relationship between weight change and CVD risk might have been confounded by health differences between those who maintained, gained or lost weight and differences in medication use across the study period. However, we adjusted for CVD risk factors including smoking, BMI, SES and cardio-protective medication use. While we adjusted for glucose-lowering medication, we were not able to separately account for metformin and sulfonylureas, as only 28 study participants were using sulfonylureas at 1 year. Sulfonylurea use was most common among participants who gained weight. Metformin use comprised the majority of glucose-lowering drug use in this cohort, reducing our concerns of bias related to differential effects of glucose-lowering drugs on weight and CVD [41]. Exclusion of participants who reported sulfonylurea use at 1 year did not change any associations (not shown). We assessed a number of risk factors at two time points, which might have increased the probability of a type I error. However, other studies have reported similar associations between weight loss and cardiovascular risk factors [42].

Of all high-risk individuals who were invited to participate in screening, 74% attended screening and all 867 individuals diagnosed with diabetes consented to enrol in the study, resulting in a study population which affords generalisability to the broader primary care population of eastern England. Due to the screening-based nature of this study, we were able to capture weight change during the period immediately following diabetes diagnosis, to assess whether moderate, achievable weight loss during this period can reduce long-term CVD risk. Adjustment for confounders including cigarette smoking and cardio-protective medication use improves the internal validity of our study relative to other studies of weight loss and CVD which did not adjust for these [11, 12].

Loss of ≥5% body weight during the year following diabetes diagnosis was associated with a lower hazard of CVD events at 10 years but was not associated with all-cause mortality. Weight loss ≥5% was also associated with improvements in HbA_1c_, diastolic BP and lipids at 1 year, while weight loss ≥10% was associated with improvements at 5 years. There is growing recognition of the impact of weight loss on diabetes remission, and our research supports the hypothesis that moderate weight loss in the year following diabetes diagnosis may yield substantial long-term CVD reduction and may be an achievable target for individuals with a new diagnosis of type 2 diabetes.

## Electronic supplementary material

ESM Tables (PDF 248 kb)

## Data Availability

The datasets generated and analysed during the current study are available from the corresponding author on reasonable request.

## Abbreviations

ADDITION–Cambridge: Anglo–Danish–Dutch Study of Intensive Treatment in People with Screen-Detected Diabetes in Primary Care–Cambridge
CVD: Cardiovascular disease
GP: General practice
Look AHEAD: Action for Health in Diabetes
MRC: Medical Research Council
NHS: National Health Service
NIHR: National Institute for Health Research
ROSE: Retrospective Study of Cardiovascular Events Related to the Use of Glucose-Lowering Drug Treatment in Primary Care
SES: Socioeconomic status
UKPDS: UK Prospective Diabetes Study
