# Supplementary material for: Moderate weight change following diabetes diagnosis and 10 year incidence of cardiovascular disease and mortality
Source: Diabetologia. 2019 May 7;62(8):1391–402. doi: 10.1007/s00125-019-4886-1 (PMC6647260; doi:10.1007/s00125-019-4886-1)
Supplement: Supplementary file 1 — (PDF 248 kb) [file 125_2019_4886_MOESM1_ESM.pdf]

Electronic Supplementary Tables

ESM Table 1. Cohort characteristics and measured CVD risk factors among adults with type 2 diabetes at time of diagnosis (baseline), 1 year, and 5 years. ADDITION-Cambridge (N=725) 2002-2014

	Female (n=279)			Male (n=446)		
Cohort characteristics	Baseline	1 year	5 years	Baseline	1 year	5 years
BMI (kg/m^2), mean (SD)	34.6 (5.9)	33.0 (5.9)	32.9 (5.8)	32.6 (5.3)	31.7 (5.1)	31.6 (5.0)
N (%) missing	1 (0.4%)	0 (0.0%)	23 (8.2%)	0 (0.0%)	0 (0.0%)	69 (15.5%)
Weight (kg), mean (SD)	88.3 (16.0)	84.1 (15.9)	83.1 (15.6)	98.6 (17.4)	95.4 (16.8)	95.0 (16.9)
N (%) missing	0 (0.0%)	0 (0.0%)	15 (5.4%)	0 (0.0%)	0 (0.0%)	38 (8.5%)
Smoking, N (%)						
Current	37 (13.3%)	31 (11.3%)	20 (8.4%)	83 (18.6%)	75 (17.0%)	51 (14.1%)
Former	104 (37.3%)	107 (38.9%)	92 (38.8%)	233 (52.2%)	236 (53.6%)	194 (53.6%)
Never	138 (49.5)	137 (49.8%)	125 (52.7%)	130 (29.2%)	129 (29.3%)	117 (32.3%)
N (%) missing	0 (0.0%)	4 (1.4%)	42 (15.1%)	0 (0.0%)	6 (1.3%)	84 (18.8)
Alcohol						
Mean units/week (SD)	3.6 (6.1)	3.0 (5.1)	3.2 (5.5)	9.9 (12.2)	8.9 (11.3)	7.0 (9.4)
N (%) missing	7 (2.5%)	8 (2.9%)	30 (10.8%)	4 (0.9%)	9 (2.0%)	67 (15.0%)
CVD Risk Factors	Baseline	1 year	5 years	Baseline	1 year	5 years
HbA_{1c}, median (Q1, Q3)						
%	6.7 (6.3, 7.5)	6.4 (6.0, 6.8)	6.8 (6.4, 7.3)	6.8 (6.2, 7.9)	6.4 (5.9, 6.9)	6.9 (6.4, 7.5)
mmol/mol	50 (45, 58)	46 (42, 51)	51 (46, 56)	51 (44, 63)	46 (41, 52)	52 (46, 58)
N (%) missing	10 (3.6%)	5 (1.8%)	17 (6.1%)	8 (1.8%)	5 (1.1%)	44 (9.9%)
Blood pressure (mmHg)						
Systolic	139.3 (19.6)	132.9 (18.4)	132.9 (15.7)	143.0 (19.6)	138.1 (18.0)	136.3 (15.8)
Diastolic	79.2 (8.9)	76.5 (8.7)	73.6 (9.4)	83.3 (10.8)	80.1 (9.8)	76.4 (9.7)
N (%) missing	2 (0.7%)	2 (0.7%)	16 (5.7%)	0 (0.0%)	0 (0.0%)	35 (7.8%)
Lipids (mmol/l)						
Total cholesterol	5.6 (1.1)	4.5 (0.9)	4.4 (0.9)	5.3 (1.1)	4.4 (1.0)	4.0 (0.8)
N (%) missing	8 (2.9%)	1 (0.4%)	17 (6.1%)	7 (1.6%)	2 (0.4%)	42 (9.4%)

HDL	1.3 (0.3)	1.4 (0.3)	1.5 (0.3)	1.1 (0.3)	1.1 (0.3)	1.2 (0.3)
N (%) missing	8 (2.9%)	1 (0.4%)	22 (7.9%)	7 (1.6%)	2 (0.4%)	52 (11.7%)
Triacylglycerols, median (Q1, Q3)	1.7 (1.2, 2.4)	1.6 (1.2, 2.2)	1.6 (1.1, 2.2)	1.8 (1.3, 2.6)	1.6 (1.2, 2.4)	1.7 (1.2, 2.4)
N (%) missing	8 (2.9%)	1 (0.4%)	21 (7.5%)	7 (1.6%)	2 (0.4%)	48 (10.8%)
Self-reported medication use, N (%)	Baseline	1 year	5 years	Baseline	1 year	5 years
Glucose-lowering						
Yes	1 (0.4%)	78 (28.8%)	155 (55.6%)	1 (0.2%)	138 (31.8%)	261 (58.5%)
No	278 (99.6%)	193 (71.2%)	124 (44.4%)	445 (99.8%)	296 (68.2%)	185 (41.5%)
Missing	0 (0.0%)	8 (2.9%)	0 (0.0%)	0 (0.0%)	12 (2.7%)	0 (0.0%)
Antihypertensive						
Yes	178 (63.8%)	198 (73.1%)	208 (74.6%)	236 (52.9%)	287 (66.1%)	316 (70.9%)
No	101 (36.2%)	73 (26.9%)	71 (25.4%)	210 (47.1%)	147 (33.9%)	130 (29.1%)
Missing	0 (0.0%)	8 (2.9%)	0 (0.0%)	0 (0.0%)	12 (2.7%)	0 (0.0%)
Lipid-lowering						
Yes	56 (20.1%)	178 (65.7%)	221 (79.2%)	117 (26.2%)	284 (65.4%)	336 (75.3%)
No	223 (79.9%)	93 (34.3%)	58 (20.8%)	329 (73.8%)	150 (34.6%)	110 (24.7%)
Missing	0 (0.0%)	8 (2.9%)	0 (0.0%)	0 (0.0%)	12 (2.7%)	0 (0.0%)

BMI: body mass index (kg/m2). CVD: cardiovascular disease. HbA_{1c}: glycated haemoglobin A_{1c}. HDL: high-density lipoprotein. mmHg: millimetre of mercury. Q1, Q3: quartile 1, quartile 3. SD: Standard deviation.

ESM Table 2. Weight at 1 year and 5 years following diabetes diagnosis, stratified by weight change during the year following diabetes diagnosis. ADDITION-Cambridge (N=725) 2002-2014.

	BMI (mean (SD))			Weight, kg (mean (SD))		
	Baseline	1 year	5 years	Baseline	1 year	5 years
Weight change category						
Gained >2%	32.3 (6.3)	33.8 (6.5)	33.3 (6.0)	91.4 (18.6)	95.7 (18.8)	93.4 (18.2)
Maintained[a]	32.8 (5.4)	32.8 (5.4)	32.1 (5.1)	94.3 (17.5)	94.0 (17.5)	91.9 (17.3)
Lost ≥2 to < 5%	33.4 (5.4)	32.4 (5.2)	32.2 (5.3)	95.0 (16.1)	91.9 (15.6)	90.3 (15.8)
Lost ≥5 to <10%	34.1 (5.1)	31.8 (4.7)	32.2 (5.2)	96.1 (16.8)	89.3 (15.6)	90.0 (17.5)
Lost ≥10%	34.2 (6.2)	29.7 (5.3)	30.8 (5.7)	94.9 (20.0)	81.7 (17.3)	85.0 (18.2)

BMI: Body mass index (kg/m^2). SD: Standard deviation.

[a]Defined as ≤2% gain or <2% loss at 1 year.

ESM Table 3. Characteristics among study participants with missing (N=137) and nonmissing (N=730) information on weight. *ADDITION-Cambridge* 2002-2014

Covariate measured at baseline, except where noted	Missing weight at baseline or 1 year (N=137)	Not missing weight (N=730)	Risk ratio[a] (missing=1)
Age (years), mean (SD)	61.1 (7.5)	61.1 (7.1)	1.00 (0.98, 1.02)
Gender (n)			
Male	82	448	0.95 (0.69, 1.30)
Female	55	282	1
Missing	0	0	
Occupational class (n)			
Professional	31	247	ref
Skilled	32	165	1.46 (0.92, 2.31)
Unskilled	70	302	1.69 (1.14, 2.50)
Missing	4	16	
Ethnicity (n)			
White	132	706	1
Black	1	3	1.59 (0.29, 8.73)
Asian	3	20	0.83 (0.28, 2.41)
Missing	1	1	
Smoking at baseline (n)			
Current	34	123	1
Former	60	339	0.69 (0.48, 1.10)
Never	62	268	0.63 (0.42, 0.94)
Missing	1	0	
Alcohol units/week, mean (SD)	8.6 (14.8)	7.5 (10.8)	1.01 (0.99, 1.02)
BMI (kg/m^2), mean (SD)	33.9 (6.3)	33.4 (5.6)	1.01 (0.98, 1.04)
Weight (kg), mean (SD)	93.6 (19.2)	94.6 (17.5)	1.00 (0.99, 1.01)
Treatment Group (n)			
Routine care	59	356	1
Intensive treatment	78	374	1.21 (0.89, 1.66)
All-cause mortality at 10 years (n)	38	99	2.05 (1.48, 2.83)
CVD incidence at 10 years (n)	22	104	1.06 (0.69, 1.64)

Type of event at 10 years (n)			
CVD death	10	21	1
MI	2	19	0.34 (0.08, 1.42)
Stroke	6	27	0.58 (0.23, 1.47)
Revascularization	4	36	0.36 (0.12, 1.09)
Amputation	0	1	n/a
HbA_{1c} (%) at baseline, mean (SD)	7.3 (1.7)	7.3 (1.7)	1.00 (0.92, 1.10)
HbA_{1c} (mmol/mol) at baseline, mean	56.3	56.3	1.00 (0.92, 1.10)
Blood pressure (mmHg), mean (SD)			
Systolic	143.2 (21.4)	141.8 (19.9)	1.00 (1.00, 1.01)
Diastolic	81.6 (11.3)	81.7 (10.3)	1.00 (0.98, 1.01)
Lipids (mmol/l), mean (SD)			
Total cholesterol	5.4 (1.2)	5.4 (1.1)	0.98 (0.85, 1.12)
LDL	3.3 (1.1)	3.3 (1.0)	1.01 (0.86, 1.19)
HDL	1.2 (0.4)	1.2 (0.3)	1.16 (0.74, 1.82)
Triacylglycerols	2.1 (2.0)	2.1 (1.5)	0.98 (0.88, 1.10)

[a]Associations between each predictor and a binary variable for missing weight value at baseline or 1 year (yes/no) were modelled using log binomial regression

ESM Table 4. Hazard ratios for the associations of change in weight in the year following diabetes diagnosis and 10-year incidence of CVD and mortality, adjusting for weight change from year 1 to year 5. *ADDITION-Cambridge* (N=725) 2002-2014

	10-year CVD incidence		10-year all-cause mortality	
Weight change	N Cases/ Total[a]	HR[b] [95% CI]	N Cases/ Total[a]	HR[b] [95% CI]
Gained >2%	4/68	0.32 [0.11, 0.92]	8/68	1.41 [0.60, 3.28]
Maintained[c]	34/157	1	14/191	1
Lost ≥2 to < 5%	23/164	0.89 [0.48, 1.65]	16/164	1.31 [0.65, 2.64]
Lost ≥5%	17/215	0.48 [0.25, 0.89]	20/215	1.17 [0.53, 2.60]

[a]Sample size where weight change, age, sex, baseline socioeconomic class, baseline BMI, smoking at 1 year, use of anti-hypertensive, lipid- or glucose-lowering medication at 1 year, and weight at 5 years are all non-missing

[b]Adjusted for age, sex, baseline socioeconomic class, baseline BMI, smoking at 1 year, weight change from year 1 to year 5, use of anti-hypertensive, lipid- or glucose-lowering medication at 1 year, and trial arm

[c]Defined as ≤2% gain or <2% loss at 1 year

ESM Table 5. Hazard ratios for the associations of change in weight in the year following diabetes diagnosis and 10 year incidence of CVD and mortality, with multiple imputation of missing information. ADDITION-Cambridge 2002-2014

	Full cohort			
	10-year CVD incidence		10-year all-cause mortality	
Weight change	N cases/ Total[a]	HR[b] [95%CI]	N cases/ Total[a]	HR[b] [95%CI]
Gained >2%	6/75	0.39 [0.17, 0.93]	13/75	1.79 [0.86, 3.72]
Maintained[c]	40/210	1	23/210	1
Lost ≥2 to < 5%	26/173	0.76 [0.46, 1.24]	22/172	1.16 [0.64, 2.11]
Lost ≥5%	22/229	0.51 [0.30, 0.86]	30/228	1.28 [0.72, 2.26]
	Age≥65 years			
	10-year CVD incidence		10-year all-cause mortality	
Gained >2%	4/21	0.50 [0.16, 1.55]	8/25	2.13 [0.82, 5.53]
Maintained[c]	20/75	1	14/75	1
Lost ≥2 to < 5%	15/71	0.82 [0.42, 1.59]	17/70	1.47 [0.71, 3.03]
Lost ≥5%	7/77	0.39 [0.17, 0.90]	14/77	1.31 [0.64, 2.70]

95% CI: 95% confidence interval. CVD: Cardiovascular disease. HR: Hazard ratio.

[a]Sample size where weight change, age, sex, baseline socioeconomic class, baseline BMI, smoking at 1 year, use of anti-hypertensive, lipid- or glucose-lowering medication at 1 year are all non-missing, prior to multiple imputation of missing information

[b]Adjusted for age, sex, baseline socioeconomic class, baseline BMI, smoking at 1 year, use of anti-hypertensive, lipid- or glucose-lowering medication at 1 year, and trial arm

[c]Defined as ≤2% gain or <2% loss at 1 year

ESM Table 6. Hazard ratios for the associations of change in weight in the year following diabetes diagnosis and 10-year incidence of CVD and mortality, among participants without self-reported myocardial infarction or stroke prior to diabetes diagnosis. *ADDITION-Cambridge* (N=648) 2002-2014

Weight change	10-year CVD incidence		10-year all-cause mortality	
	N Cases/ Total[a]	HR[b] [95% CI]	N Cases/ Total[a]	HR[b] [95% CI]
Gained >2%	3/64	0.23 [0.05, 1.15]	8/64	1.24 [0.52, 2.96]
Maintained[c]	33/183	1	19/183	1
Lost ≥2 to < 5%	23/155	0.80 [0.44, 1.48]	16/155	1.07 [0.55, 2.11]
Lost ≥5%	15/212	0.40 [0.23, 0.70]	25/212	1.17 [0.55, 2.46]

95% CI: 95% confidence interval. CVD: Cardiovascular disease. HR: Hazard ratio.
[a]Sample size where weight change, age, sex, baseline socioeconomic class, baseline BMI, smoking at 1 year, use of anti-hypertensive, lipid- or glucose-lowering medication at 1 year are all non-missing
[b]Adjusted for age, sex, baseline BMI, smoking at 1 year, socioeconomic class, use of anti-hypertensive, lipid- or glucose-lowering medication at 1 year, and trial arm
[c]Defined as ≤2% gain or <2% loss at 1 year.

ESM Table 7. Hazard ratios for the association of change in weight in the year following diabetes diagnosis and 10-year incidence of myocardial infarction, stroke, and CVD death. *ADDITION-Cambridge* 2002-2014

Weight change	N Cases/ Total[a]	HR[b] [95% CI]
Gained >2%	4/71	0.39 [0.10, 1.52]
Maintained[c]	25/185	1
Lost ≥2 to < 5%	15/158	0.74 [0.35, 1.57]
Lost ≥5%	14/215	0.51 [0.27, 0.96]

[a]Sample size where weight change, age, sex, baseline socioeconomic class, baseline body mass index, smoking at 1 year, use of anti-hypertensive, lipid- or glucose-lowering medication at 1 year, are all non-missing

[b]Hazard ratio adjusted for age, sex, baseline socioeconomic class, baseline body mass index, smoking at 1 year, use of anti-hypertensive, lipid- or glucose-lowering medication at 1 year, and trial arm

[c]Defined as ≤2% gain or <2% loss at 1 year.

ESM Table 8. Subdistribution hazard ratios for the association of change in weight in the year following diabetes diagnosis and 10-year incidence of CVD. *ADDITION-Cambridge* 2002-2014

Weight change	N Cases/ Total[a]	sHR[b] [95% CI]
Gained >2%	6/75	0.41 [0.16, 1.03]
Maintained[c]	40/210	1
Lost ≥2 to < 5%	26/173	0.78 [0.47, 1.30]
Lost ≥5%	22/229	0.54 [0.31, 0.93]

[a]Sample size where weight change, age, sex, baseline socioeconomic class, baseline body mass index, smoking at 1 year, use of anti-hypertensive, lipid- or glucose-lowering medication at 1 year, are all non-missing

[b]Subdistribution hazard ratio adjusted for age, sex, baseline socioeconomic class, baseline body mass index, smoking at 1 year, use of anti-hypertensive, lipid- or glucose-lowering medication at 1 year, and trial arm

[c]Defined as ≤2% gain or <2% loss at 1 year.

ESM Table 9. Beta coefficients from multivariable linear regression models of the associations of change in weight in the year following diabetes diagnosis and CVD risk factors measured 1 and 5 years after diagnosis, with multiple imputation of missing information. *ADDITION-Cambridge* 2002-2014

	Risk factors measured at 1 year	Risk factors measured at 5 years
	β[a] [95% CI]	β[a] [95% CI]
HbA_{1c} (mmol/mol)		
Gained >2%	2.19 [-0.37, 4.74]	-0.73 [-3.74, 2.27]
Maintained[b]	0	0
Lost ≥2% to <5%	-2.18 [-4.00, -0.35]	-1.39 [-3.75, 0.98]
Lost ≥5% to <10%	-4.83 [-6.83, -2.83]	-1.11 [-3.79, 1.58]
Lost ≥10%	-6.87 [-9.31, -4.43]	-3.57 [-6.46, -0.67]
Systolic BP (mmHg)		
Gained >2%	1.29 [0.24,2.35]	-1.11 [-2.00,-0.22]
Maintained[b]	0	0
Lost ≥2 to < 5%	-3.06 [-3.86,-2.25]	-1.76 [-2.42,-1.09]
Lost ≥5 to <10%	-1.93 [-2.80,-1.05]	1.20 [0.47,1.93]
Lost ≥10%	-2.99 [-3.96,-2.02]	-1.95 [-2.76,-1.14]
Diastolic BP (mmHg)		
Gained >2%	0.05 [-0.49,0.59]	-0.47 [-1.00,0.06]
Maintained[b]	0	0
Lost ≥2 to < 5%	-1.08 [-1.49,-0.66]	-0.33 [-0.72,0.07]
Lost ≥5 to <10%	-1.93 [-2.38,-1.48]	-0.39 [-0.83,0.04]
Lost ≥10%	-2.88 [-3.37,-2.38]	-1.86 [-2.34,-1.38]
Total Cholesterol (mmol/l)		
Gained >2%	-0.27 [-0.32,-0.22]	-0.11 [-0.15,-0.06]
Maintained[b]	0	0
Lost ≥2 to < 5%	-0.08 [-0.12,-0.04]	-0.16 [-0.19,-0.12]
Lost ≥5 to <10%	-0.20 [-0.25,-0.16]	-0.13 [-0.17,-0.09]
Lost ≥10%	-0.28 [-0.32,-0.23]	-0.17 [-0.21,-0.13]
LDL (mmol/l)		
Gained >2%	-0.22 [-0.42,-0.02]	-0.05 [-0.25,0.15]

Maintained[b]	0	0
Lost ≥2 to < 5%	-0.05 [-0.20,0.09]	-0.19 [-0.33,-0.06]
Lost ≥5 to <10%	-0.09 [-0.25,0.08]	-0.11 [-0.26,0.04]
Lost ≥10%	-0.12 [-0.28,0.05]	-0.14 [-0.31,0.03]
Triacylglycerols[c](mmol/l)		
Gained >2%	-0.29 [-0.37,-0.22]	-0.29 [-0.34,-0.23]
Maintained[b]	0	0
Lost ≥2 to < 5%	-0.31 [-0.37,-0.25]	-0.12 [-0.17,-0.08]
Lost ≥5 to <10%	-0.49 [-0.56,-0.43]	-0.14 [-0.19,-0.09]
Lost ≥10%	-0.84 [-0.91,-0.77]	-0.43 [-0.48,-0.38]

95% CI: 95% confidence interval. BP: Blood pressure. CVD: Cardiovascular disease. HbA_{1c}: Glycated haemoglobin A_{1c}. HDL: High-density lipoprotein. LDL: Low-density lipoprotein.

[a]Beta coefficients are from linear regression models adjusted for age, sex, baseline BMI, smoking, relevant medication use, and trial arm.

[b]Defined as ≤2% gain or <2% loss at 1 year.

[c]Natural log of triacylglycerols.

ESM Table 10. Beta coefficients and 95% confidence intervals for the associations of weight change in the year following diabetes diagnosis and CVD risk factors at one and 5 years after diagnosis, adjusting for baseline risk factors. *ADDITION-Cambridge* (N=725) 2002-2014.

	Risk factors measured at 1 year		Risk factors measured at 5 years	
**Risk factor**	N	β^a (95% CI)	N	β^a (95% CI)
HbA_{1c} (mmol/mol)				
Gained >2%	70	1.04 [-1.49, 3.56]	72	-1.48 [-4.15, 1.18]
Maintained[b]	176	0	187	0
Lost ≥2% to <5%	159	-1.68 [-3.62, 0.26]	165	-1.27 [-3.43, 0.90]
Lost ≥5% to <10%	124	-4.81 [-6.41, -3.21]	127	-1.03 [-3.72, 1.66]
Lost ≥10%	92	-6.42 [-8.76, -4.08]	94	-3.59 [-6.04, -1.15]
Systolic BP				
Gained >2%	70	1.80 [-3.73,7.33]	72	-0.87 [-5.20,3.46]
Maintained[b]	189	0	203	0
Lost ≥2 to < 5%	164	-0.64 [-3.61,2.34]	171	-0.69 [-4.37,2.99]
Lost ≥5 to <10%	127	-2.73 [-6.46,1.00]	130	0.58 [-3.23,4.39]
Lost ≥10%	94	-4.60 [-8.97,-0.25]	96	-3.78 [-8.15,0.59]
Diastolic BP				
Gained >2%	70	-0.09 [-2.05,1.86]	72	-0.58 [-3.67,2.50]
Maintained[b]	189	0	203	0
Lost ≥2 to < 5%	164	-0.31 [-1.73,1.11]	171	-0.26 [-2.04,1.52]
Lost ≥5 to <10%	127	-2.06 [-3.54,-0.58]	130	-0.68 [-2.45,1.09]
Lost ≥10%	94	-2.97 [-4.99,-0.95]	96	-2.54 [-4.55,-0.52]
Total cholesterol				
Gained >2%	71	-0.26 [-0.51,0.00]	72	-0.07 [-0.24,0.11]
Maintained[b]	182	0	194	0
Lost ≥2 to < 5%	159	-0.12 [-0.27,0.03]	163	-0.18 [-0.32,-0.043]
Lost ≥5 to <10%	124	-0.24 [-0.45,-0.03]	125	-0.11[-0.26,0.04]
Lost ≥10%	93	-0.37 [-0.53,-0.22]	96	-0.23[-0.39,-0.08]
LDL				
Gained >2%	67	-0.20[-0.42,0.01]	66	-0.03 [-0.20,0.14]

Maintained[b]	167	0	176	0
Lost ≥2 to < 5%	150	-0.08[-0.21,0.04]	148	0.21 [-0.35,-0.07]
Lost ≥5 to <10%	120	-0.10 [-0.27,0.07]	116	-0.11 [-0.241,0.02]
Lost ≥10%	90	-0.18 [-0.31,-0.04]	99	-0.19 [-0.33,-0.04]
Triacylglycerols[c]				
Gained >2%	71	-0.04 [-0.18,0.11]	71	-0.12 [-0.22,-0.01]
Maintained[b]	181		191	0
Lost ≥2 to < 5%	159	-0.09 [-0.17,-0.02]	161	-0.07 [-0.15,0.01]
Lost ≥5 to <10%	124	-0.21 [-0.30,-0.13]	123	-0.08 [-0.19,0.02]
Lost ≥10%	93	-0.41 [-0.51,-0.32]	94	-0.27 [-0.36,-0.18]

95% CI: 95% confidence interval. BP: Blood pressure. CVD: Cardiovascular disease. HbA_{1c}: Glycated haemoglobin A_{1c}. HDL: High-density lipoprotein. LDL: Low-density lipoprotein.

[a]Beta coefficients are from linear regression models adjusted for age, sex, baseline BMI, smoking, relevant medication use, trial arm, and baseline value of the risk factor.

[b]Defined as ≤2% gain or <2% loss at 1 year.

[c]Natural log of triacylglycerols

ESM Table 11. Beta coefficients from multivariable linear regression models of the associations of change in weight in the year following diabetes diagnosis and CVD risk factors measured 1 and 5 years after diagnosis, by medication use. *ADDITION-Cambridge* (N=725) 2002-2014

	Stratified by relevant medication use at 1 year				Stratified by relevant medication use at 5 years			
	N	Yes medication use at 1 year β[a] [95% CI]	N	No medication use at 1 year β[a] [95% CI]	N	Yes medication use at 5 years β[a] [95% CI]	N	No medication use at 5 years β[a] [95% CI]
HbA_{1c} (mmol/mol)								
Gained >2%	31	2.83 [-2.28, 7.93]	39	1.48 [-1.61, 4.57]	27	1.09 [-2.44, 4.61]	37	-2.94 [-7.17, 1.29]
Maintained[b]	62	0	123	0	56	0	112	0
Lost ≥2% to <5%	60	-3.30 [-6.37, -0.23]	104	-1.96 [-4.21, 0.29]	56	-2.51 [-5.78, 0.76]	98	1.11 [-1.68, 3.91]
Lost ≥5% to <10%	41	-6.81 [-9.73, -3.88]	84	-4.15 [-6.23, -2.07]	38	-3.39 [-7.20, 0.42]	80	2.35 [-1.02, 5.72]
Lost ≥10%	21	-6.35 [-12.45, -0.24]	71	-6.15 [-8.37, -3.93]	21	-4.32 [-8.47, -0.16]	66	-1.03 [-3.48, 1.43]
Systolic BP (mmHg)								
Gained >2%	55	1.37 [-5.61,8.35]	15	2.44 [-9.68,14.56]	56	0.72 [-4.14,5.59]	16	-9.35 [-17.59,-1.11]
Maintained[b]	136	0	53	0	159	0	44	0
Lost ≥2 to < 5%	128	-2.48 [-6.92,1.96]	36	-4.41 [-10.80,1.98]	133	-2.37 [-6.31,1.57]	39	-1.92 [-7.05,3.20]
Lost ≥5 to <10%	92	-2.33 [-7.52,2.86]	35	-0.46 [-7.28,6.37]	97	2.71 [-2.39,7.81]	33	-3.29 [-10.31,3.73]
Lost ≥10%	71	-3.06 [-8.31,2.18]	23	-0.98 [-7.96,6.00]	69	-0.36 [-5.14,4.43]	27	-8.53 [-19.07,2.00]
Diastolic BP (mmHg)								
Gained >2%	55	-0.56 [-3.04,1.93]	15	3.05 [-2.08,8.18]	57	0.26 [-3.20,3.73]	21	-3.07 [-8.61,2.46]
Maintained[b]	136	0	53	0	162	0	60	0
Lost ≥2 to < 5%	128	-1.26 [-3.39,0.87]	36	-0.76 [-3.67,2.14]	135	-0.92 [-3.02,1.17]	46	0.21 [-3.80,4.22]
Lost ≥5 to <10%	92	-1.96 [-3.60,-0.33]	35	-1.36 [-4.74,2.01]	98	-0.27 [-2.27,1.72]	40	-1.70 [-5.98,2.58]

Lost ≥10%	71	-3.48 [-6.26,-0.71]	23	-0.53 [-3.49,2.43]	70	-2.21 [-4.86,0.44]	33	-2.73 [-7.83,2.37]
Total Cholesterol (mmol/l)								
Gained >2%	51	-0.32 [-0.58,-0.50]	20	-0.19 [-0.73,0.34]	62	-0.13 [-0.32,0.06]	17	0.11 [-0.37,0.59]
Maintained[b]	122	0	66	0	163	0	58	0
Lost ≥2 to < 5%	128	-0.15 [-0.37,0.06]	37	0.11 [-0.23,0.46]	144	-0.17 [-0.32,-0.02]	38	-0.15 [-0.57,0.26]
Lost ≥5 to <10%	94	-0.17 [-0.41,0.07]	32	-0.26 [-0.75,0.24]	109	-0.06 [-0.26,0.14]	28	-0.40 [-0.90,0.10]
Lost ≥10%	65	-0.25 [-0.44,-0.05]	28	-0.31 [-0.69,0.07]	76	-0.13 [-0.31,0.04]	26	-0.29 [-0.81,0.23]
LDL (mmol/l)								
Gained >2%	51	-0.25 [-0.50,0.00]	20	-0.19 [-0.64,0.27]	59	-0.03 [-0.25,0.18]	10	0.12 [-0.37,0.62]
Maintained[b]	122	0	66	0	154	0	40	0
Lost ≥2 to < 5%	128	-0.08 [-0.26,0.10]	37	0.05 [-0.30,0.41]	138	-0.15 [-0.31,0.01]	30	-0.26 [-0.63,0.11]
Lost ≥5 to <10%	94	-0.02 [-0.22,0.19]	32	-0.17 [-0.57,0.23]	106	-0.06 [-0.22,0.10]	19	-0.23 [-0.698,0.24]
Lost ≥10%	65	-0.04 [-0.21,0.12]	28	-0.16 [-0.51,0.18]	75	-0.09 [-0.27,0.09]	19	-0.26 [-0.74,0.23]
Triacylglycerols[c] (mmol/l)								
Gained >2%	51	-0.08 [-0.26,0.11]	20	-0.14 [-0.37,0.08]	61	-0.15 [-0.32,0.02]	10	-0.33 [-0.72,0.05]
Maintained[b]	122	0	66	0	156	0	41	0
Lost ≥2 to < 5%	128	-0.09 [-0.22,0.04]	37	-0.16 [-0.32,0.01]	138	-0.08 [-0.21,0.05]	30	-0.05 [-0.28,0.19]
Lost ≥5 to <10%	94	-0.21 [-0.34,-0.08]	32	-0.20 [-0.34,-0.06]	107	-0.05 [-0.23,0.12]	18	-0.26 [-0.48,-0.05]
Lost ≥10%	65	-0.48 [-0.64,-0.32]	28	-0.38 [-0.61,-0.14]	74	-0.26 [-0.43,-0.09]	20	-0.34 [-0.55,-0.13]

[a]Beta coefficients are from linear regression models adjusted for age, sex, baseline BMI, smoking, relevant medication use at 1 year or 5 years, and trial arm.

[b]Defined as ≤2% gain or <2% loss at 1 year.

[c]Natural log of triacylglycerols.
